# Measurement of skin surface dose distributions in radiation therapy using poly(vinyl alcohol) cryogel dosimeters

**DOI:** 10.1002/acm2.12087

**Published:** 2017-04-24

**Authors:** Molham M. Eyadeh, Marcin Wierzbicki, Kevin R. Diamond

**Affiliations:** ^1^ Physics Department Faculty of Science Yarmouk University Irbid Jordan; ^2^ Department of Medical Physics and Applied Radiation Sciences Juravinski Cancer Centre McMaster University Hamilton ON Canada

**Keywords:** cryogel, PVA, radiation dosimeter, radiation therapy, radiochromic bolus dosimeter, skin surface dose, superficial dose

## Abstract

In external beam radiation therapy (EBRT), skin dose measurement is important to evaluate dose coverage of superficial target volumes. Treatment planning systems (TPSs) are often inaccurate in this region of the patient, so *in vivo* measurements are necessary for skin surface dose estimation. In this work, superficial dose distributions were measured using radiochromic translucent poly(vinyl alcohol) cryogels. The cryogels simultaneously served as bolus material, providing the necessary buildup to achieve the desired superficial dose. The relationship between dose to the skin surface and dose measured with the bolus was established using a series of oblique irradiations with gantry angles ranging from 0° to 90°. EBT‐2 Gafchromic film was placed under the bolus**,** and the ratio of bolus‐film dose was determined ranging from 0.749 ± 0.005 to 0.930 ± 0.002 for 0° and 90° gantry angles, respectively. The average ratio over 0–67.5° (0.800 ± 0.064) was used as the single correction factor to convert dose in bolus to dose to the skin surface. The correction factor was applied to bolus measurements of skin dose from head and neck intensity‐modulated radiation therapy (IMRT) treatments delivered to a RANDO phantom. The resulting dose distributions were compared to film measurements using gamma analysis with a 3%/3 mm tolerance and a 10% threshold. The minimum gamma pass rate was 95.2% suggesting that the radiochromic bolus may provide an accurate estimation of skin surface dose using a simple correction factor. This study demonstrates the suitability of radiochromic cryogels for superficial dose measurements in megavoltage photon beams.

## 1 Introduction

In EBRT, the dose deposited at the skin surface by megavoltage photon beams is definitively lower than that deposited within underlying tissues due to a lack of electronic equilibrium. However, treatment of superficial disease, such as skin lesions or shallow lymph nodes, requires that the prescribed dose be delivered up to the skin surface. A layer of bolus may be placed on the skin to increase electron fluence, increasing the dose deposited in the superficial tissues.^1^, ^2^, ^3^, ^4^, ^5^, ^6^ Other factors influencing the surface dose distribution include electron contamination from the linear accelerator, obliquity, field size, beam modifiers, air gap, and delivery technique.^7^, ^8^, ^9^, ^10^, ^11^, ^12^, ^13^, ^14^ Accurate knowledge of dose to superficial tissues is necessary to ensure that shallow targets receive the prescribed dose while the dose to normal tissue is within tolerance.^15^, ^16^ However, this is confounded due to the inaccuracy of most TPSs in the buildup region.^17^

Modern radiotherapy TPSs are able to calculate skin dose within ±25%.^18^, ^19^, ^20^, ^21^, ^22^, ^23^ Most TPSs estimate skin surface dose by extrapolating measured data with fitting functions.^24^, ^25^ Monte Carlo simulation is capable of calculating the dose in the buildup region accurately^26^, ^27^, ^28^ but the use of these systems is limited in the clinic due to the computational requirements.^29^ Therefore, *in vivo* measurements are desirable to verify the skin surface dose.

Several dosimeters are currently used in radiotherapy for surface skin dose estimation. Thermoluminescent detectors (TLDs),^14^, ^23^, ^30^, ^31^, ^32^ diodes,^33^, ^34^ and metal oxide semiconductor field effect transistors (MOSFETs)^29^, ^35^, ^36^, ^37^ may be used to produce low resolution surface dose distributions. Radiographic or radiochromic film may be used to quantitate the distribution of surface dose in two dimensions.^38^, ^39^, ^40^, ^41^ Radiochromic film has several advantages, such as tissue equivalency, self development, and high spatial resolution.^42^ However, film is difficult to form to surfaces that contain both convex and concave regions, which complicates dosimetry.^40^ In these situations, a more flexible material is desirable.

Gel dosimeters may be used to measure skin dose at individual points with small fields and steep dose gradients treatments such as dosimetry of IMRT and stereotactic radiosurgery. Such techniques involve the delivery of a high radiation dose using small size radiation beams.^43^, ^44^, ^45^ Gel dosimeters such as cryogels are flexible and can easily conform to the skin over large, complex, curved regions. Furthermore, as suggested by Chu et al.^46^ without further investigation, poly(vinyl alcohol) cryogels (PVA‐C) based dosimeters may be simultaneously employed as a dosimetric bolus to provide an accurate estimation of skin surface dose. PVA‐C is flexible and stable, and, when loaded with a radiosensitive material such as ferrous benzoic xylenol orange (FBX), is capable of recording dose in two and three dimensions.^46^, ^47^ These cryogels may be used as both a buildup material and act as an *in vivo* dosimeter to monitor the treatment delivery. These cryogels were used to monitor chest wall radiation therapy treatment; it can quantitate uncertainties in setup, and breathing irregularities during left breast or chest wall deep inspiration breathing hold (DIBH) technique.^48^, ^49^ And therefore, the PVA‐C‐based dosimeter may used as a dosimetric bolus for simultaneous skin dose boosting and measurement during radiotherapy to provide an accurate estimation of superficial dose distribution.

The dose estimated using TPS at depths of 0.5–1.0 cm lacks the accuracy typically desired for radiotherapy targets. Thus, a dosimetric bolus material would be useful in simultaneously increasing dose to superficial targets and in ensuring these areas receive the prescribed dose. For megavoltage photon beams, the dose increases up to 60% within the first 0.5 cm depth, making the measured surface dose sensitive to the buildup thickness.^25^, ^29^, ^36^ Due to the rapid dose build up, the dose will not be uniform throughout the 0.5 cm thick bolus material, and not equal to the actual surface dose. In this work, a correction factor is determined from the dose measured in the gel dosimeter to the surface dose.

Eyadeh et al. (2014)^50^ described a FBX‐PVA‐C material that may be used as radiochromic bolus readable in two dimensions using a simple camera system. The electron density of the FBX‐PVA‐C material is 1.05 gm/cm^3^, which is almost water equivalent. The material is translucent, allowing visualization of underlying skin marks to assist in patient and beam positioning. The cryogel dosimeter is deformable with good stability and sensitivity, no significant dose rate or energy dependence for this dosimeter; the cryogel signals are constant after 2 hr post exposure with 2.8 × 10^−4^ mm^−1^ cGy^−1^ rate of base line drift post exposure.^50^ The purpose of this work was to evaluate the ability of FBX‐PVA‐C radiosensitive bolus material to measure skin dose during radiotherapy. The concept is generated using clinical IMRT fields delivered to the head and neck region of a RANDO phantom (Phantom Laboratory, Salem, NY, USA).

## 2 Materials and methods

### 2.A. Translucent FBX PVA‐C dosimeter preparation

In this study, translucent FBX‐PVA‐C was used as radiochromic bolus. A detailed description of its production was described elsewhere.^50^ Briefly, PVA concentration of 15% by weight was selected to optimize sensitivity, fabrication time, sturdiness, and ease of handling of the finished slabs of bolus.

The main ingredients of the formulation were all acquired from Sigma Aldrich, St. Louis, MO, USA. The components were 99% hydrolyzed PVA (molecular weight 146–188 kDa), dimethyl sulfoxide ≥ 99% (DMSO), ferrous ammonium sulphate (ammonium iron (II) sulphate hexahydrate, 99%), benzoic acid ≥ 99.5%, and xylenol orange (XO) tetrasodium salt. Sulphuric acid of 95–98% purity was also used.

The PVA was dissolved in 25 mM sulphuric acid, water, and DMSO at 120°C (70 mL of 20 water/80 DMSO by weight). The hydrogel was cooled to 50°C, at which point a 10 mL solution of 0.55 mM ferrous ammonium sulphate, 5 mM benzoic acid, and 1 mM XO tetrasodium salt, all dissolved in 25 mM sulphuric acid, was added to the hydrogel. A solution of 25 mM sulphuric acid water/DMSO was stirred in during the final 10 min to make up the desired total volume (100 mL). The mixture was then evacuated for 15 min to remove any visible air bubbles.

The hydrogel was decanted into custom plastic molds with interior dimensions of 15 × 15 cm² and 0.5 cm thickness, the error in the thickness was approximately 1% or less for all cryogel samples. The hydrogels were subjected to three cycles of 18 hour freezing at −80°C and 6 hr thawing at room temperature. The finished cryogels were removed from their molds and cut to size as necessary. The cryogel is flexible and can conform to most parts of a patient's body.

### 2.B Radiochromic bolus read out apparatus

**Figure 1:**
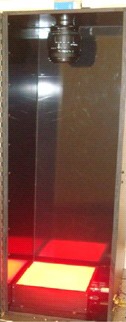
In‐house 2D optical imaging apparatus. The lens is 61 cm away from the LED array, and the light box is 15 × 15 cm^2^. Excess area on the light surface was masked using black construction paper to improve the dynamic range of the system.

The radiochromic bolus samples were imaged pre‐ and post irradiation using the equipment shown in Fig. 1. A template was used to ensure reproducible placement of the radiochromic bolus. The apparatus was composed of a 1392 × 1024 pixel charge coupled device (CCD) camera (Nikon Corporation, Tokyo, Japan), a 28–105 mm, f/1.4–5.6, a (UC‐II) zoom lens (Sigma Corporation, Fukushima, Japan), a Lumen‐Essence BK‐600 uniform red light emitting diode (LED) array (Luminus Devices Inc., Billerica, MA, USA), housed in a light tight box. The lens was focused on the center of the radiochromic bolus to optimize the resolution. The images captured by the CCD were transferred to a computer, and stored as 16‐bit “TIFF gray image” files. The noise in the images was reduced through post processing using the “wdencmp” algorithm native to the MATLAB image‐processing toolbox (MathWorks Inc., Natick, MA, USA). The 2D linear absorption coefficients maps (i.e., per mm) were computed using in‐house Matlab code. All measurements and irradiations were performed at room temperature; post irradiation imaging was performed 2 hr after irradiation, and the premeasurements were acquired 10 min before irradiation.

### 2.C Calibration of radiochromic bolus and film

The relationship between bolus absorption coefficient and delivered dose was established using a Varian iX linear accelerator (Varian Inc., Palo Alto, CA, USA) with 6 MV photons and 20 × 20 cm^2^ field size. The 0.5 cm thick samples of 7 × 7 cm^2^ radiochromic bolus were sandwiched at isocenter between two 5.6 cm slabs of polystyrene. Doses ranging from 100 to 4000 cGy were applied with a dose rate of 633 cGy/min. The expected doses in cGy for this arrangement were computed using the Pinnacle v9.2 TPS (Philips, Amsterdam, Netherlands).

The same procedure was employed to relate optical density and dose in 7 × 7 cm^2^ pieces of EBT‐2 Gafchromic film (lot #A052810‐01) (International Specialty Products, Wayne, NJ, USA). Doses ranging from 100 to 1500 cGy were applied with a dose rate of 633 cGy/min. The expected doses in the film were also computed with Pinnacle 9.2. As described later, film was used as the gold standard measurement of skin dose.

The process of film marking, read out, and analysis was consistent with the manufacturer's recommendations.^51^ EBT‐2 film was also read prior to irradiation to obtain the background optical density; the net optical density of each irradiated film was obtained by subtracting the background optical density. The EBT‐2 film was scanned using an Epson 11000XL Scanner (Proscan, Avision, Australia) and analyzed using Film QA^TM^ Pro (Ashland, Wayne, NJ, USA). The described film calibration curve above was used for the film response. The red channel data were used during film analysis at a resolution of 150 DPI; color correction was disabled. All films were read out approximately 24 hr after their irradiation. Calibration irradiations and subsequent read out were performed at room temperature. Subsequent comparisons of radiochromic bolus and film were also performed in the Film QA software suite.

### 2.D Calibration of radiochromic bolus for skin surface dosimetry

**Figure 2:**
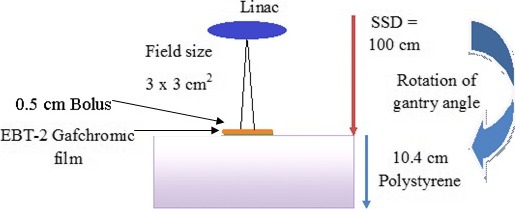
Schematic of the radiochromic bolus and EBT‐2 film irradiation. A 3 × 3 cm² field was formed using the jaw collimator and 1000 MU were delivered with a rate of 600 MU/min. The procedure was repeated with gantry rotations ranging from 0° to 90°.

Open field irradiations with normal and oblique incidence were used to examine the relationship between the dose distribution recorded by the radiochromic bolus and the true surface dose, which was estimated using EBT‐2 film. The configuration of these measurements is shown in Fig. 2. A 0.5 cm thick, 7 × 7 cm^2^ radiochromic bolus sample and 7 × 7 cm^2^ piece of EBT‐2 film were stacked on the surface of a 10.4 cm slab of polystyrene. The film was positioned at isocenter, 100 cm away from the source. The irradiation was repeated with gantry angles of 0°, 22.5°, 45°, 67.5°, and 90°. At each angle, a 3 × 3 cm² field was formed using the jaw collimator and 1000 monitor units (MU) were delivered with a rate of 600 MU/min. The irradiations of films were also planned in Pinnacle to compute the surface dose distribution.

Optical density and absorption coefficient measured in film and bolus, respectively, were converted to doses using the respective calibration curves. Finally, the ratio between the film and radiochromic bolus measured dose distributions was obtained to serve as a correction factor for scaling radiochromic bolus dose to dose on the skin surface.

### 2.E Validation of skin suface dosimetry using radiochromic bolus

A Philips Brilliance Big Bore scanner was used to acquire 1 mm thick CT slices of the head and neck portion of a RANDO phantom. The CT data were exported to Pinnacle 9.2. Two simple static parallel‐opposed‐pair (POP) beam arrangements were planned for the neck region; two previously treated clinical cases with bolus including a three‐field larynx and a nine‐field head and neck IMRT were selected and positioned on the RANDO data to approximate the arrangement on the patient. The use of the TPS in this part of the study was to locate and contour custom bolus for the various field arrangements: virtual bolus was drawn on the RANDO CT images to approximate the shape and size of the clinically used bolus material. These plans were then delivered to the phantom with EBT‐2 film placed on the phantom surface below a layer of 0.5 cm radiochromic bolus to evaluate the skin surface dose directly. The bolus was manually cut to size on the phantom surface using orthogonal light field projections as a guide.

**Figure 3:**
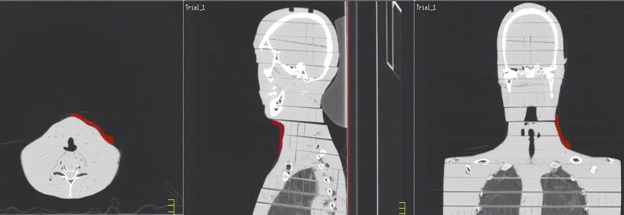
An example of radiochromic bolus definition. The bolus is 0.5 cm thick.

The two tangential POP static beams (0°/180° and 90°/270°) were delivered to the 0.5 cm radiochromic bolus and film stack on the surface of the RANDO phantom. The beams were 3 × 3 cm^2^ open fields static beam arrangements delivered to the neck with 6 MV photons and 250 MU with a rate of 600 MU/min. Figure 3 shows the placement of the radiochromic bolus.

The two clinical step‐and‐shoot IMRT plans were delivered as closely to the original planned conditions as possible. The larynx treatment included three step‐and‐shoot fields, with a total of 276 MU delivered at 400 MU/min. The head and neck treatment employed nine fields to deliver multiple dose levels to superficial disease and various neck nodes with total of 593 MU at 400 MU/min.

## 3 Results and discussion

### 3.A Calibration of radiochromic bolus for skin surface dosimetry

**Figure 4:**
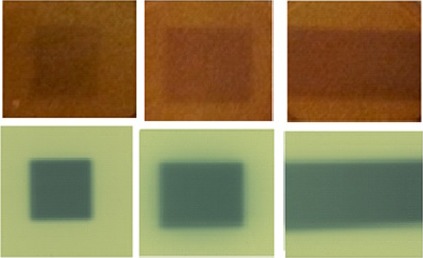
Photographs of irradiated radiochromic bolus and EBT‐2 film arising from the configuration shown in Fig. 2: gantry angles of 0°, 45°, and 90° from left to right.

The relationship between absorption coefficient and dose delivered to the 0.5 cm slabs of radiochromic bolus in its linear range was (3.00 ± 0.04) × 10^−4^ mm^−1^ cGy^−1^, which is consistent with our previous measurements.^49^, ^50^ Figure 4 shows the irradiated radiochromic bolus and EBT‐2 film following exposures of 1000 MU at gantry angles of 0°, 45°, and 90°.

Measurements correlating the dose distribution in bolus to the expected distribution at the underlying surface (estimated using Gafchromic EBT‐2 film) indicated that surface dose increased with gantry angle. Dose increases because beam enters obliquely and experiences a larger path length. This is consistent with previous investigations that reported increasing surface doses with incident beam angles with a rapid increase beyond about 45° incidence.^14^, ^52^, ^53^

**Figure 5:**
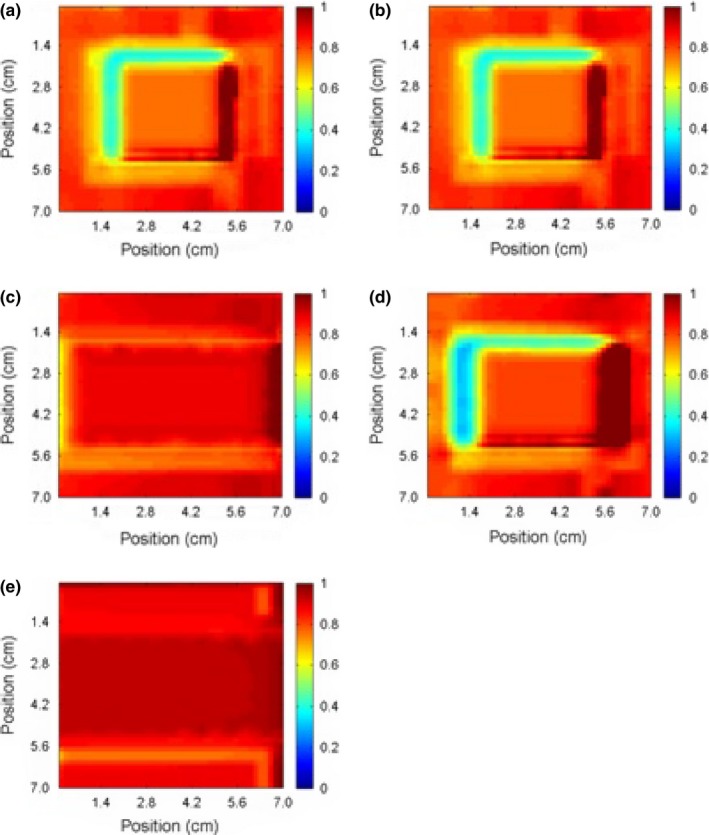
The ratio of the measured dose distribution in a radiochromic bolus to the surface dose distribution estimated using Gafchromic EBT‐2 film for gantry angles of (a) 0°, (b) 22.5°, (c) 45°, (d) 67.5°, and (e) 90°.

Film QA Pro software (Ashland, Wayne, NJ, USA) was used to visually align the radiochromic bolus and film dose maps. The point‐by‐point ratio was computed from these aligned images and is shown in Fig. 5. The mean ratios for the irradiated areas are summarized in Table 1. The goal of measuring these ratios was to develop a simple approach to convert dose in bolus to dose on skin. A simpler approach would be to use a single correction factor independent of the angle of incidence. The range of ratios shown in Table 1 seems to suggest this approach may not be feasible. However, if we restrict ourselves to a range of angles, 0°–67.5° for example, we find that the average ratio between radiochromic bolus and film is 0.800 ± 0.064. Using this correction factor, the agreement between the film and the corrected bolus was improved at all gantry angles. This factor was used to correct all subsequent bolus images.

**Table 1:** The mean ratio between surface dose and the dose measured in the radiochromic bolus at different gantry angles ranging from 0° to 90°

Gantry angle (°)	Mean ratio ± standard deviation
0	0.749 ± 0.005
22.5	0.760 ± 0.005
45	0.802 ± 0.009
67.5	0.890 ± 0.016
90	0.930 ± 0.002

The mean ratio between radiochromic bolus and film for normal incidence can be estimated by integrating over the percent depth dose (PDD) data (e.g., Varian Golden Beam data) and dividing by the dose at the underlying surface (in this case, depth 0.5 cm). For the linear accelerator used in this study, the predicted dose ratio was 0.739, which agrees well with the measured value of 0.749 ± 0.005.

For all gantry angles in the POP exposures, the TPS overestimated the surface dose measured with film by 14.3–25.6%. This is consistent with related studies with various dosimeters; Dogan and Glasgow (2003) observed that the TPS overestimated surface dose by 25% compared to a parallel plate ion chamber measurement.^19^ Chung et al. reported that two TPSs overestimated surface dose by up to 18.5% when compared to Gafchromic film measurements.^20^ Court et al. showed that the agreement between TPS calculated skin surface dose was within 20% of doses measured using MOSFETs.^21^ Kry et al. (2011) reported an overall 22% difference in surface dose between TPS and TLDs.^23^ These types of discrepancies arise in model‐based TPSs due to challenges in modeling electron contamination and regions of electronic disequilibrium.^25^

**Figure 6:**
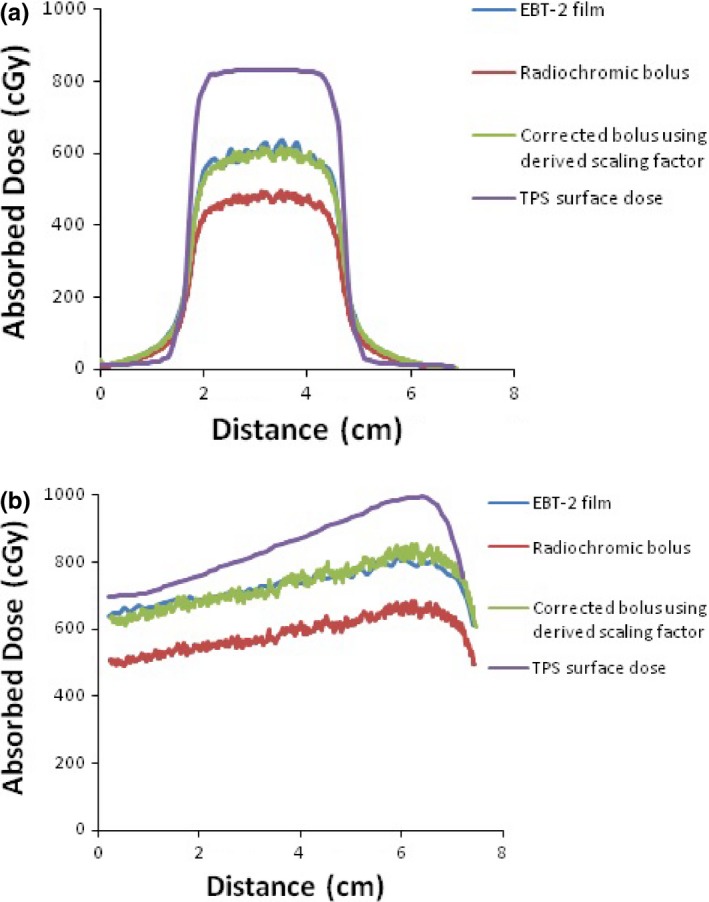
Crossline profiles (*x*‐axis) of absolute dose distributions extracted from Gafchromic film, radiochromic bolus, corrected radiochromic bolus using the derived scaling factor of 0.80, and TPS surface dose for (a) 0° and (b) 90° gantry angles.

**Figure 7:**
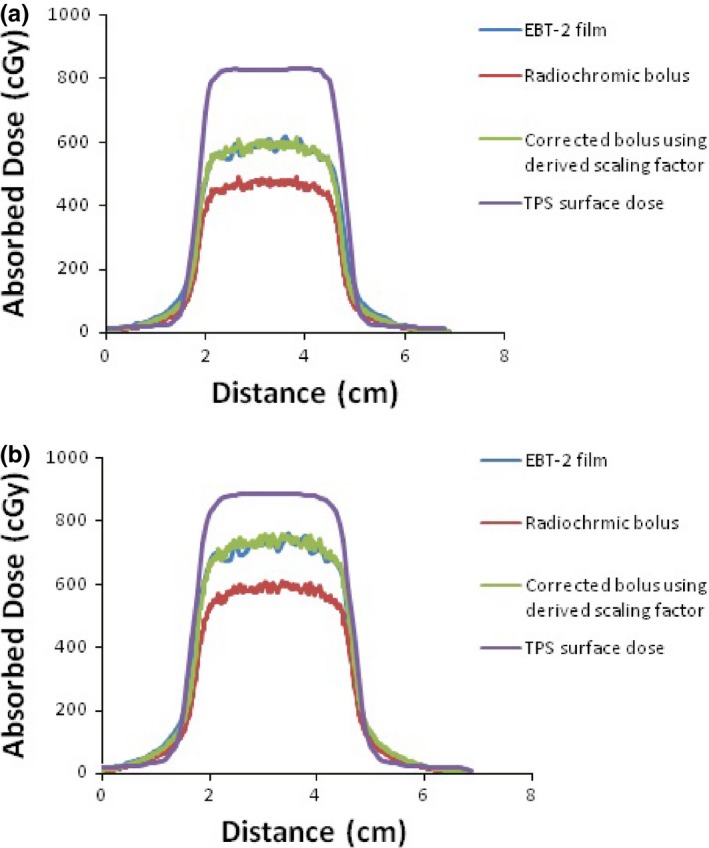
Central inline profiles (*y*‐axis) of absolute dose distributions extracted from Gafchromic film, radiochromic bolus, corrected radiochromic bolus using the derived scaling factor of 0.80, and TPS surface dose for (a) 0° and (b) 90° gantry angles.

With the application of the correction factor, good agreement of inline and crossline profiles between the corrected bolus and Gafchromic film was observed at all gantry angles, with average differences ranging from 1.4 to 1.9%. As an example, the central inline (*y*‐axis) and crossline (*x*‐axis) axes of the absolute dose distributions measured at 0° and 90° gantry angles with Gafchromic film, radiochromic bolus, and corrected radiochromic bolus using the derived scaling factor of 0.800 are shown in Figs. 6 and 7, respectively. The corresponding dose profiles were calculated using the TPS, and these are also shown in Figs. 6 and 7.

This suggests that a 0.5 cm radiochromic cryogel should be able to predict dose deposited at the bolus‐skin interface. Although the irradiation geometry was quite simple, it was dosimetrically more challenging than realistic treatment geometries where exit dose from some beams may help to mitigate the large differences in buildup seen due to beam obliquity.

### 3.B Validation of skin surface dosimetry using radiochromic bolus

The correction factor of 0.800 was applied to the radiochromic bolus measurements for the four treatment plans. This correction factor was applied to head and neck region treatment plans contain variations in filed size and SSD.

**Figure 8:**
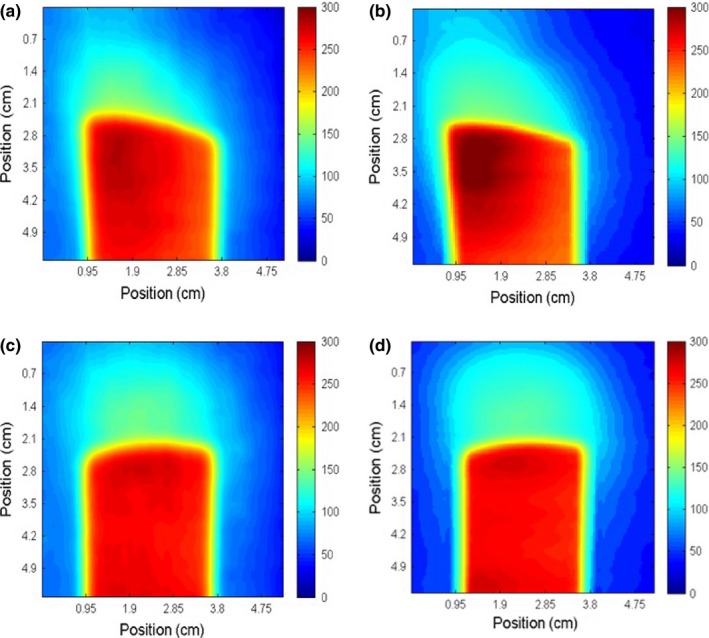
Absorbed dose in cGy measured with radiochromic bolus (a, c) and Gafchromic EBT‐2 film (b, d) for two tangential POP static beams of (0°/180°) and (90°/270°) respectively.

**Figure 9:**
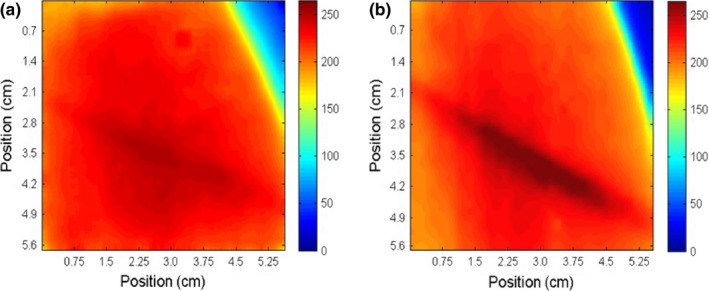
Absorbed dose in cGy measured with radiochromic bolus (panel a) and Gafchromic EBT‐2 film (panel b) for step‐and‐shoot IMRT larynx treatment.

**Figure 10:**
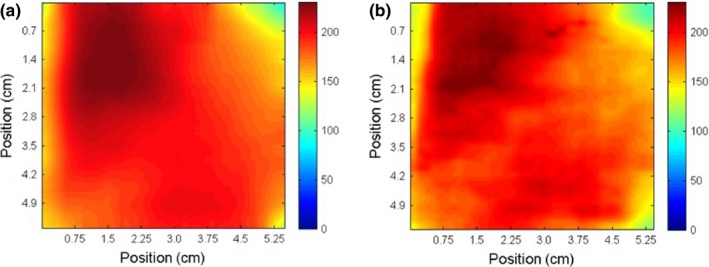
Absorbed dose in cGy measured with radiochromic bolus (panel a) and Gafchromic EBT‐2 film (panel b) for step‐and‐shoot IMRT neck treatment.

Figure 8 compares the absolute dose distributions of the POP beams measured using radiochromic bolus and film. Measurements of the larynx plan are shown in Fig. 9, and measurements of the neck plan are shown in Fig. 10.

Gamma analysis is typically used to judge the agreement between treatment plan and dose measurements.^54^, ^55^ In our study, it was used to evaluate the agreement between the corrected radiochromic bolus and EBT‐2 film measurements. A 2D gamma analysis was performed using the Film QA Pro software using 3%/3 mm criteria and a 10% dose threshold. The bolus distribution was used as the reference for the gamma evaluation. The passing rate was obtained from the generated gamma maps where a failing pixel was assigned a value greater than 1 and a passing pixel was assigned values between 0 and 1. The percentage of pixels passing the gamma evaluation ranged from 95.2 to 96.4% as shown in Table 2.

**Table 2:** Gamma pass rates for comparisons of corrected radiochromic bolus and Gafchromic film measurements (3%/3 mm, 10% threshold) for different field arrangements

Irradiation geometry	Gamma passing rate (%)
0°/180° POP, static beams	96.1
90°/270° POP, static beams	96.4
3‐field beams larynx, IMRT	95.2
9‐field beams neck, IMRT	95.5

POP, parallel‐opposed‐pair; IMRT, intensity‐modulated radiation therapy.

The above results suggest that the radiochromic bolus measures skin dose with sufficient accuracy for clinical use. The disagreement between corrected bolus and film is around the level of uncertainty in film dosimetry of approximately 3%.^25^, ^35^, ^40^, ^41^, ^56^ Furthermore, the FBX PVA‐C material is more flexible than film, providing improved skin contact. It can be wrapped easily around curved surfaces and provide *in vivo* dosimetry in areas where skin dose quantitation is desired. In addition, it is often difficult to place the standard bolus without air gaps on the surface; PVA‐FBX‐C is flexible enough to provide skin contact without air gap due to its rubbery and adhesive nature. Our system employs a simple calibration process and one correction factor for accurate skin dose estimation for the head and neck region. Different kinds of treatments including other locations on the body with different cryogel thicknesses may yield different results and would have to be tested. However, for head and neck treatments with a typical bolus thickness the results of this paper should be valid.

While the use of radiochromic cryogel bolus for *in vivo* dosimetry is viable, it may not be practical in some cases, especially if large quantities of material are desired. The cryogels used in this study were produced over a 3 day period using a −80°C freezer, although variations can be produced over a 24 hr period using a standard food freezer operated at −20°C. The required manufacturing lead time may introduce workflow challenges. Our previous work has demonstrated that this particular formulation of radiochromic cryogel is stable for at least 1 week prior to irradiation, suggesting that bolus could be manufactured in advance and stored for short periods of time.^50^ Optical read out of the cryogels may be performed using relatively simple equipment: a camera with full manual control and a spatially uniform light source that overlaps spectrally with, in this case, the Xylenol Orange absorption peak.

## 4 Conclusions

Skin dosimetry is an important aspect in radiotherapy of superficial targets considering that TPSs are inaccurate in this region of the patient. Radiochromic bolus shows potential as an *in vivo* dosimetry tool, where it may be used to accumulate a dose distribution and read out immediately after the treatment delivery. A comparison of EBT‐2 Gafchromic film and FBX‐PVA‐C radiochromic bolus suggests that the latter may provide an accurate estimation of skin surface dose distribution using a simple correction factor. Radiochromic bolus may then be used in place of more traditional forms of bolus to perform *in vivo* dosimetry in regions where the skin dose is important. The main advantage of this system over film is that it is more flexible, allowing it to be wrapped around complex curved surfaces. It may be possible to improve agreement between radiochromic bolus and EBT‐2 film using a more complex, angle dependent correction scheme, but this may overcomplicate the dose estimation process. In this study, the dose distributions recorded in the corrected cryogels and film were not compared with the Pinnacle skin dose, as this requires projecting a flattened 2D dose image onto the original 3D surface. It may be necessary to evaluate the correction factor for different parts of the body. For example, quantitating skin dose in tangential irradiation of the chest wall, where the beams are quite oblique to the skin surface. However, we feel that it is feasible to use a radiochromic cryogel as an *in vivo* dosimeter to evaluate superficial dose distributions.
